# Association between neutrophilic granulocyte percentage and depression in hospitalized patients with heart failure

**DOI:** 10.1186/s12888-016-1161-6

**Published:** 2016-12-13

**Authors:** Shuo Pan, Zhong-Wei Liu, Ying Lv, Wen-Qian Song, Xun Ma, Gong-Chang Guan, Yong Zhang, Shun-Ming Zhu, Fu-Qiang Liu, Bo Liu, Zhi-Guo Tang, Jun-Kui Wang

**Affiliations:** 1 First Department of Cardiology, People’s Hospital of Shaanxi Province, Xi’an, 710068 Shaanxi People’s Republic of China; 2 Department of Internal Medicine, University Hospital of Northwest University, Xi’an, Shaanxi People’s Republic of China; 3 Department of Emergency Medicine, People’s Hospital of Shaanxi Province, Xi’an, Shaanxi People’s Republic of China

**Keywords:** Neutrophilic granulocyte percentage, Depression, Hospitalized heart failure

## Abstract

**Background:**

Previous researches reveal that depression is associated with increased inflammatory markers. As a simple and cheap inflammatory marker, we hypothesize that neutrophilic granulocyte percentage is associated with depression in hospitalized heart failure patients, whose prevalence of depression is at a very high level.

**Methods:**

Three hundred sixty-six cases of hospitalized heart failure patients with left ventricular ejection fraction (LVEF) ≤45% and New York Heart Association (NYHA) class II-IV were enrolled. All the enrolled patients received Hamilton Rating Scale for Depression (24-items) (HAM-D_24_). The demographic, clinical data, blood samples and echocardiography were documented. The Pearson simple linear correlation was performed to evaluate the confounding factors correlated with HAM-D_24_ depression index. The significantly correlated factors were enrolled as independent variables in Logistic regression to determine the risk or protective factors for depression, which was taken as dependent variable.

**Results:**

Two hundred ten cases of hospitalized heart failure patients (57.4%) had depression. Among them, 134 patients (63.8%) had mild depression, 58 patients (27.6%) had moderate depression and 18 patients (8.6%) had severe depression. Pearson simple linear correlation revealed that in hospitalized patients with heart failure, the neutrophils granulocyte percentage was positively correlated with the HAM-D_24_ depression index (*r* = .435, *p* < .001). After the adjustment of age, BMI, number of members of the household, smoking index, New York Heart Association (NYHA) classification, hemoglobin, TC, LDL-C, creatinine, cystatin-C, TBIL and albumin, the neutrophils granulocyte percentage is still significantly associated with depression in hospitalized heart failure patients (*OR* = 1.046, *p* < .001).

**Conclusions:**

The neutrophils granulocyte percentage may be used as a new marker for depression in hospitalized heart failure patients.

## Background

Heart failure is the end stage of all cardiovascular diseases, it mainly shows symptoms of dyspnea and edema, which severely affect exercise capacity and quality of life. Heart failure is prevalent around the globe, an estimated 5.7 million Americans have heart failure [1], and there are at least 15 million patients with heart failure in Europe [2]. Eleven million patients suffered from heart failure in the year 2010 in China [3]. Heart failure is one major cause of death and disability. Even though extensive pharmacologic and device therapeutics were developed for heart failure, the morbidity and mortality is still high in heart failure patients [4].

Depression is common in heart failure, with a reported prevalence of 21.5% in a meta-analysis [5]. However, the depression may be still under-diagnosed with the highest prevalence of up to 77.5% in one cohort study [6]. Depression is one of many factors associated with poor prognosis in heart failure patients [7–9]. It has been reported to be independently associated with poor quality of life, limited functional status, and increased risk of morbidity and mortality in this population [10–14].

Previous researches reveal that depression is associated with increased inflammatory markers including C-reactive protein (CRP), interleukin-1, and interleukin-6 [15–17]. One study reports that the increased neutrophil/lymphocyte ratio is correlated with the severity of depression in patients with depression [18]. As one of the important indicator of inflammation, neutrophilic granulocyte percentage is cheap and simple test contained in blood routine test. We hypothesized that such an easily attained test result may be have certain association with depression and be used in predicting the depression in highly depressed heart failure patients. So far, the association between the neutrophilic granulocyte percentage and depression were not reported in such a population. Therefore, the aim of the present study is to investigate the association between the neutrophilic granulocyte percentage and depression in hospitalized heart failure patients.

## Methods

### Subjects

We performed this present cross-sectional and observational study in 1^st^ Cardiology Department of People’s Hospital of Shaanxi Province from January 2015 to June in 2016. We enrolled hospitalized patients with left ventricular ejection fraction (LVEF) ≤45% and New York Heart Association (NYHA) class II-IV heart failure symptoms. New York Heart Association (NYHA) class II is defined as mild symptoms (mild shortness of breath and/or angina) and slight limitation during ordinary activity. New York Heart Association (NYHA) class III is defined as marked limitation in activity due to symptoms, even during less-than-ordinary activity, comfortable only at rest. New York Heart Association (NYHA) class IV is defined as severe limitations in activity, even while at rest, who are mostly bedbound patients [19]. Heart failure was confirmed by clinical heart failure specialist and cardiologists after consulting with the echocardiography report. The main exclusion criteria included significant cognitive impairment, alcohol or drug dependence within the previous year, psychoses, bipolar disorder, severe personality disorder, active suicidal ideation, life-threatening comorbidity, and current use of antipsychotic or antidepressant medications [20].

Three hundred eighty-five hospitalized patients with heart failure were screened, a total 19 patients were excluded. 8 persons were excluded as they were taking alprazolam and diazepam, 3 persons were excluded since they were heavy alcohol users, 4 persons were excluded as they had severe cognitive impairment after stroke, 2 persons were excluded as they suffered continuous dyspea and edema till death, 2 persons were excluded as they are not willing to cooperate. Among the enrolled 366 hospitalized patients, 200 (54.6%) patients were male and 166 (45.4%) patients were female. The average age was 62.56 ± 10.74 years.

### Depression assessments

The heart failure patients were evaluated for depressive symptoms by using Hamilton Rating Scale for Depression (24-items) (HAM-D_24_), it contained a total of 24 items (10 item was defined from 0 to 2, and 14 items were defined from 0 to 4). Questions of 0–2 points were defined as none (0), mild-moderate (1), severe (2). Questions of 0–4 points were defined as none (0), mild (1), moderate (2), severe (3), very severe (4). HAM-D_24_ score <8 points was defined as non-depression and HAM-D_24_ score ≥8 points was defined as depression. HAM-D_24_ score of 8–19 points was defined as mild depression, HAM-D_24_ score of 20–34 points was defined as moderate depression, HAM-D_24_ score of ≥35 points was defined as severe depression [21]. The measurements of HAM-D_24_ were conducted by trained physicians on the first day after admission. Two physicians in our department were sent to psychology department for a week to learn the talking, observing and interpreting skill of HAM-D_24_. The two physicians independently scores for each patients, the mean scores taken by the physicians were used as the final score. If the two scores were severely diverted, the repeated test will be performed by a psychologist in psychology department.

### Demographic and clinical data

Demographic data and cardiovascular risk factors were obtained from the medical records. Body weight was measured while the subjects were without shoes by using a double balance placed on a firm surface. Height was measured by using a Frankfort plane positioned at a 90° angle against a wall-mounted metal tape. The waist circumference measurements were taken at the end of normal expiration and to the nearest 0.1 cm, measuring from the narrowest point between the lower borders of the rib cage and the iliac crest [22].

### Blood samples and echocardiography

Peripheral blood was sampled from patients in a fasting state on the morning following the admission day. Venous blood samples were sent to Clinical Laboratory Department of People’s Hospital of Shaanxi Province for red blood cells (RBC) counts, hemoglobin, platelet counts, plateletcrit, mean platelet volume (MPV), platelet distribution width (PDW), white blood cells (WBC) counts, neutrophilic granulocyte percentage (NEUT%), total cholesterol (TC), triglyceride, high-density lipoprotein-cholesterol (HDL-C), low-density lipoprotein-cholesterol (LDL-C), apolipoprotein A, apolipoprotein B, urea nitrogen (BUN), creatinine, cystatin-C, total bilirubin (TBIL), direct bilirubin (DBIL), total protein (TP), albumin, brain natriuretic peptide (BNP) and fasting glucose (FG) detection using standard biochemical techniques. Echocardiographic data (left ventricular ejection fraction [LVEF]) was obtained using Doppler echocardiography conducted within 3 days of admission [23].

### Definition of risk factors

Hypertension was defined as an average systolic blood pressure ≥ 140 mmHg, or an average diastolic blood pressure ≥ 90 mmHg, or both, or self-reported use of antihypertensive medication, or a self-reported history of hypertension.

Diabetes was defined as fasting plasma glucose ≥ 7.0 mmol/L, or random plasma glucose ≥ 11.1 mmol/L, or 2 h plasma glucose in oral glucose tolerance test (OGTT) ≥ 11.1 mmol/L, or use of insulin or oral hypoglycemic agents, or a self-reported history of diabetes.

Coronary artery disease was defined as the presence of at least one significant coronary artery stenosis of more than 50% luminal diameter in coronary angiography or coronary computed tomographic angiography (CTA).

Atrial fibrillation was diagnosed mainly via electrocardiogram (ECG), characteristic findings included absence of P waves and irregular R-R intervals.

Dilated cardiomyopathy was diagnosed mainly via echocardiogram, which showed left ventricular dilatation with normal or thinned walls and reduced ejection fraction. Meanwhile, the history of coronary artery disease, hypertension, valvular heart disease and other heart diseases should be excluded before making the present diagnosis.

Smoking index was defined as number of cigarettes smoked per day × years of smoking.

BMI was calculated as weight in kg divided by height in m^2^.

### Statistical analysis

The statistical analysis was conducted using SPSS version 16.0 for Windows (SPSS Inc., Chicago, IL, USA). Continuous variables were expressed as mean ± standard deviations and the differences between the depression group and non-depression group were analyzed using the Mann-Whitney *U*-test. Categorical variables were expressed as proportions and the differences in categorical variables were analyzed using chi-square test. Pearson correlation analysis was conducted to determine the correlation between HAM-D_24_ score and each clinical and laboratory factor. Logistic regression model was established to determine whether neutrophilic granulocyte percentage was significantly associated with depression after the adjustment of each confounding factor. Statistical significance was established at *p* < 0.05.

## Results

The baseline characteristics of hospitalized heart failure patients with and without depression were shown in Table 1. Two hundred ten patients developed depression accounting for 57.4% of all hospitalized patients with heart failure. Among them, 134 patients (63.8%) had mild depression, 58 patients (27.6%) had moderate depression and 18 patients (8.6%) had severe depression. 31.3% of male patients had depression while 68.7% of female patients had depression, the distribution of depression between men and women was statistically significant. The distribution of depression in each New York Heart Association (NYHA) classification also varied significantly, the depression rates increased dramatically in patients with New York Heart Association (NYHA) class III and class IV. The incidence of coronary artery disease and diabetes mellitus showed significant differences between the two groups. The incidence of diabetes was higher in heart failure patients with depression while the incidence of coronary artery disease was lower in heart failure patients with depression when compared with that in heart failure patients without depression. The smoking index, platelet counts, plateletcrit, NEUT%, TC, triglyceride, LDL-C, apolipoprotein B, BUN, creatinine, cystatin-C, BNP and HAM-D_24_ score were significantly higher in heart failure patients with depression when compare with those without depression. Meanwhile, the waist circumference, monthly family income and hemoglobin and left ventricular ejection fraction (LVEF) were significantly lower in heart failure patients with depression when compare with those without depression. The habitant area, the incidence of hypertension, atrial fibrillation and dilated cardiomyopathy, age, BMI, number of members of the household, RBC counts, MPV, PDW, WBC counts, HDL-C, apolipoprotein A, TBIL, DBIL, TP, albumin and FG showed no significant difference between the heart failure patients with depression and ones without depression.

**Table 1:** Baseline characteristics of hospitalized heart failure patients with and without depression

	Without depression (*n* = 156)	With Depression (*n* = 210)	*p* value
Sex			<.001
Men	104 (52.0%)	96 (31.3%)	
Women	52 (48.0%)	114 (68.7%)	
Habitant area			.069
Urban area	108 (46.2%)	48 (36.4%)	
Rural area	126 (53.8%)	84 (63.6%)	
NYHA classification			<.001
II	144 (92.3%)	122 (58.1%)	
III	8 (5.1%)	68 (32.4%)	
IV	4 (2.6%)	20 (9.5%)	
Coronary artery disease	138 (88.5%)	164 (78.1%)	.010
Hypertension	90 (57.7%)	116 (55.2%)	.640
Atrial fibrillation	26 (16.7%)	48 (22.9%)	.145
Dilated cardiomyopathy	8 (5.1%)	18 (8.6%)	.205
Diabetes mellitus	42 (26.9%)	78 (37.1%)	.039
Age (years)	62.55 ± 9.56	62.58 ± 11.61	.723
BMI (kg/m^2^)	23.82 ± 2.73	23.46 ± 4.33	.404
Waist circumference (cm)	86.18 ± 8.71	84.16 ± 10.29	.017
Monthly family income (yuan)	3869.23 ± 3118.49	3019.05 ± 1782.27	.003
Number of members of the household (person)	4.04 ± 1.27	3.88 ± 1.35	.199
Smoking index	73.97 ± 203.39	100.14 ± 190.25	<.001
RBC counts (×10^12^/L)	4.35 ± 0.49	4.21 ± 0.60	.091
Hemoglobin (g/L)	135.24 ± 16.05	131.00 ± 18.37	.035
Platelet counts (×10^9^/L)	176.54 ± 45.62	190.26 ± 58.96	.018
Plateletcrit (%)	0.18 ± 0.05	0.19 ± 0.05	.021
MPV (fL)	10.33 ± 1.48	10.30 ± 1.31	.954
PDW (fL)	16.45 ± 2.28	16.24 ± 2.05	.127
WBC counts (×10^9^/L)	6.93 ± 5.00	6.54 ± 1.82	.285
NEUT% (%)	30.69 ± 32.60	63.31 ± 12.13	<.001
TC (mmol/L)	3.95 ± 0.88	4.67 ± 1.13	<.001
Triglyceride (mmol/L)	1.40 ± 0.69	1.76 ± 1.18	.015
HDL-C (mmol/L)	1.19 ± 0.28	1.18 ± 0.36	.390
LDL-C (mmol/L)	1.87 ± 0.60	2.26 ± 0.77	<.001
Apolipoprotein A (g/L)	1.14 ± 0.21	1.14 ± 0.27	.748
Apolipoprotein B (g/L)	0.72 ± 0.19	1.22 ± 3.47	<.001
BUN (mmol/L)	5.82 ± 2.51	6.65 ± 3.50	.046
Creatinine (umol/L)	73.98 ± 24.26	85.63 ± 40.81	.001
Cystatin-C (mg/L)	1.09 ± 0.32	1.27 ± 0.54	.001
TBIL (umol/L)	17.12 ± 8.90	18.63 ± 15.39	.913
DBIL (umol/L)	5.94 ± 3.13	6.67 ± 6.94	.410
TP (g/L)	64.04 ± 5.70	63.08 ± 6.15	.134
Albumin (g/L)	38.34 ± 5.47	37.51 ± 4.91	.343
BNP (pg/mL)	364.15 ± 832.65	601.65 ± 986.63	<.001
FG (mmol/L)	6.63 ± 1.54	7.66 ± 2.78	.148
LVEF (%)	39.29 ± 3.25	35.59 ± 6.94	<.001
HAM-D_24_ score	4.32 ± 1.67	17.58 ± 8.36	<.001

*NYHA* New York Heart Association, *BMI* body mass index, *RBC* red blood cells, *MPV* mean platelet volume, *PDW* platelet distribution width, *WBC* white blood cells, *NEUT%* neutrophilic granulocyte percentage, *TC* total cholesterol, *HDL-C* high-density lipoprotein-cholesterol, *LDL-C* low-density lipoprotein-cholesterol, *BUN* urea nitrogen, *TBIL* total bilirubin, *DBIL* direct bilirubin, *TP* total protein, *BNP* brain natriuretic peptide, *FG* fasting glucose, *LVEF* left ventricular ejection fraction

Pearson correlation analysis between HAM-D_24_ score and clinical and laboratory factors in hospitalized heart failure patients were shown in Table 2. The age, number of members of the household, smoking index, New York Heart Association (NYHA) classification, NEUT%, TC, LDL-C, BUN, creatinine, cystatin-C, TBIL, DBIL and BNP showed significantly positive correlation with HAM-D_24_ score in hospitalized heart failure patients. The correlation coefficient between NEUT% and HAM-D_24_ score was 0.435 (*p* < .001), which was the second highest correlation coefficient with HAM-D_24_ in all the confounding factors. Meanwhile, the BMI, RBC counts, hemoglobin, albumin and left ventricular ejection fraction (LVEF) showed significantly negative correlation with HAM-D_24_ score in hospitalized heart failure patients. The waist circumference, monthly family income, platelet counts, plateletcrit, MPV, PDW, WBC counts, triglyceride, HDL-C, apolipoprotein A, apolipoprotein B, TP, FG showed no significant correlation with HAM-D_24_ score in hospitalized heart failure patients.

**Table 2:** Pearson correlation analysis between HAM-D_24_ score and clinical and laboratory factors in hospitalized heart failure patients

	*r*	*p* value
Age	.104	.046*
BMI	-.106	.043*
Waist circumference	-.076	.149
Monthly family income	-.076	.147
Number of members of the household	.143	.006*
Smoking index	.103	.048*
NYHA classification	.603	<.001*
RBC counts	-.189	<.001*
Hemoglobin	-.146	.007*
Platelet counts	-.044	.420
Plateletcrit	-.049	.369
MPV	.009	.864
PDW	.019	.730
WBC counts	-.097	.074
NEUT%	.435	<.001*
TC	.326	<.001*
Triglyceride	.057	.286
HDL-C	-.053	.328
LDL-C	.354	<.001*
Apolipoprotein A	-.089	.099
Apolipoprotein B	.008	.878
BUN	.162	.003*
Creatinine	.190	<.001*
Cystatin-C	.269	<.001*
TBIL	.150	.005*
DBIL	.137	.011*
TP	-.068	.210
Albumin	-.175	.001*
BNP	.243	<.001*
FG	.090	.524
LVEF	-.271	<.001*

*HAM-D*_*24*_Hamilton rating scale for depression (24 items), *BMI* body mass index, *NYHA* New York Heart Association, *RBC* red blood Cells, *MPV* mean platelet volume, *PDW* platelet distribution width, *WBC* white blood cells, *NEUT%* neutrophilic granulocyte percentage, *TC* total cholesterol, *HDL-C* high-density lipoprotein-cholesterol, *LDL-C* low-density lipoprotein-cholesterol, *BUN* urea nitrogen, *TBIL* total bilirubin, *DBIL* direct bilirubin, *TP* total protein, *BNP* brain natriuretic peptide, *FG* fasting glucose, *LVEF* left ventricular ejection fraction; *P < 0.05

Logistic regression analysis for HAM-D_24_ determined depression using neutrophilic granulocyte percentage and each confounding factor in hospitalized heart failure patients were presented in Table 3. We enrolled all the factors significantly correlated with HAM-D_24_ score except for RBC counts, BUN, DBIL, BNP and left ventricular ejection fraction (LVEF) in the Logistic regression. Since the highly tight correlations were found between hemoglobin and RBC counts, BUN and creatinine, DBIL and TBIL, New York Heart Association (NYHA) classification and BNP, left ventricular ejection fraction (LVEF) in clinical practices, we considered it inappropriate if we put all the highly correlated factors in one Logistic regression model. Therefore, we chose the hemoglobin, creatinine, TBIL and New York Heart Association (NYHA) classification in the Logistic regression model rather than RBC counts, BUN, DBIL, BNP and left ventricular ejection fraction (LVEF). After the adjustment of age, BMI, number of members of the household, smoking index, New York Heart Association (NYHA) classification, hemoglobin, TC, LDL-C, creatinine, cystatin-C, TBIL and albumin, NEUT% is still significantly associated with depression in hospitalized heart failure patients (*OR* = 1.046, *p* < .001).

**Table 3:** Logistic regression analysis for HAM-D_24_ determined depression using neutrophilic granulocyte percentage and each confounding factor in hospitalized heart failure patients

	*Odd ratios*	95% *CI*	*p* value
Age	0.988	0.959–1.018	.420
BMI	0.996	0.914–1.086	.933
Number of members of the household	0.945	0.754–1.184	.622
Smoking index	1.000	0.998–1.002	.830
NYHA classification	2.757	1.361–5.586	.005*
Hemoglobin	0.987	0.967–1.007	.191
NEUT%	1.046	1.029–1.063	<.001*
TC	2.038	1.213–3.424	.007*
LDL-C	0.942	0.459–1.933	.871
Creatinine	0.996	0.976–1.016	.703
Cystatin-C	1.589	0.420–6.017	.495
TBIL	0.994	0.966–1.023	.693
Albumin	0.952	0.897–1.011	.111

*HAM-D*_*24*_ Hamilton rating scale for depression (24 items), *CI* confidence interval, *BMI* body mass index, *NYHA* New York Heart Association, *NEUT%* neutrophilic granulocyte percentage, *TC* total cholesterol, *LDL-C* low-density lipoprotein-cholesterol, *TBIL* total bilirubin; *P < 0.05

## Discussions

The present study revealed that correlation coefficient between NEUT% and HAM-D_24_ score was 0.435 (*p* < .001). Then the Logistic regression model confirmed that NEUT% is still associated with depression in hospitalized heart failure patients with the adjustment of age, BMI, number of members of the household, smoking index, New York Heart Association (NYHA) classification, hemoglobin, TC, LDL-C, creatinine, cystatin-C, TBIL and albumin (*OR* = 1.046, *p* < .001). The mechanism that the NEUT% was associated with depression in heart failure patients is still unclear. The possible reason may be as follows. As an important indicator for inflammation, the neutrophilic granulocytes would be activated firstly when responding to inflammation exposure. The activated neutrophilic granulocytes, which can be interpreted as increased NEUT% may be accompanied with elevated inflammatory cytokines secretion causing heavier oxidative stress and further inflammation. Meanwhile, some studies [24, 25] found that in patients with severe depressive disorder, the activation of proinflammatory cytokines and inhibition of interferon-gamma, interleukin-2, and interleukin-4 were documented. Therefore, we believe that the activation of inflammation may play the key role in the association with depression. The heart failure may amplify the association via the increase of inflammation and oxidative stress. However, the causal relationship between the inflammation and depression needs to be further clarified.

The present study showed that the depression rates increased dramatically in patients with New York Heart Association (NYHA) class III and class IV, we also found that the predictors of severity of heart failure, such as New York Heart Association (NYHA) classification, BNP, and EF were all significantly correlated with HAM-D_24_ score (all *p* < .001). The correlation coefficients were 0.603 for New York Heart Association (NYHA) classification, −0.271 for left ventricular ejection fraction (LVEF) and 0.243 for BNP, the New York Heart Association (NYHA) classification was proved to be a risk factor after adjustment of other confounding factors (*OR* = 2.757, *p* = .005). Relevant research has also shown a higher prevalence of depression in patients with more severe heart failure [26, 27]. Pena et al. [28] showed that heart failure patients in New York Heart Association (NYHA) grade IV were more depressed than those in New York Heart Association (NYHA) grades II or III. Gottlieb et al. [6] demonstrated that patients classified as New York Heart Association (NYHA) class III and IV were more likely to be depressed than class II patients. In their meta-analysis, Rutledge et al. [5] demonstrated that higher prevalencerates were associated with worse New York Heart Association (NYHA) class. Polikandrioti et al. [4] found that higher levels of depression were observed for heart failure patients in New York Heart Association (NYHA) II and NYHA III compared to those in grade I.

In this present study, we found that the traditional risk factors for heart failure were significantly correlated with HAM-D_24_ score. A part of the reason may be that anemia (counts and hemoglobin), dyslipidemia (TC and LDL-C), kidney dysfunction (BUN, creatinine and cystatin-C) and liver dysfunction (TBIL, DBIL and albumin) were classical features accompanied with heart function deterioration, as the heart function decreased, the symptoms of heart failure began to appear, which may cause the depression in this population [29–32]. Besides these traditional factors, the present study revealed 4 demographic factors significantly correlated with HAM-D_24_ score. First, HAM-D_24_ score increased as patients got elder, considering that the cardiovascular diseases were highly age related, it is not difficult to understand that depression level will rise with increase of age. Second, BMI would decrease as the increase of depression level, a majority of heart failure patients had sodium and water retention, remission of sodium and water retention will lead to some weight loss, which will improve symptoms of heart failure and depression [33]. Third, larger number of members of the household was correlated with more depression. This was an interesting Chinese phenomenon that the more number of members of the household did not mean more family support and less loneliness, it meant more argument and relatively less space in the room for each person. Fourth, smoking index significantly increased as HAM-D_24_ score increased. It was also an interesting phenomenon that smoking may not necessarily bring you happiness and relief, it made you more depressed if you had heart failure. The reason may be that smoking activated the process of arteriosclerosis making you more vulnerable to the harm of cardiovascular disease [34].

By using HAM-D_24_ score, a convenient and accurate tool for the depression evaluation [35, 36], the present study showed that 57.4% of all hospitalized patients with heart failure had depression. The prevalence of depression in heart failure patients varied dramatically in previous studies. Rutledge et al. [5] reported the prevalence of depression as 21.5% in a meta-analytic review. Rafanelli et al. [37] who studied both hospitalized patients and outpatients with heart failure, showed that 38.1% of the sample experienced depression. One study in Greek population [38] showed that 41.6% of hospitalized patients with heart failure appeared to have depression. Gottlieb et al. [6] reported depression rates among hospitalized patients of 13 to 77.5% and rates for outpatients from 13 to 42%. Vaccarino et al. [39] showed that 78% of hospitalized heart failure patients experienced depressive symptoms. These differences in the prevalence of depression in heart failure patients are possibly attributable to differences in enrollment criteria, diagnostic instruments, the definition and classification of depression, and the severity of heart failure. This highlights the importance of using a universally accepted measuring instrument that will allow comparisons between populations.

Our study has several strengths. First, we provide clinical and laboratory data in hospitalized heart failure patients divided by depression and the relationship between the confounding factors and depression, which conveyed valuable clue for other studies searching new risk factors for depression in hospitalized heart failure patients. Second, we have shown for the first time the association between NEUT% and depression in hospitalized heart failure patients. Third, the blood routine test is a simple, cheap and commonly used test, so NEUT% can be easily acquired. NEUT% would present us with new insight into the depression status in hospitalized heart failure patients.

Our study has several limitations. This study was a cross-sectional and observational study, the causal relationship between NEUT% and depression need to be further clarified. Further cohort studies may provide treatment to patients with depression and then evaluate whether the NEUT% showed the decrease trend after the depression was alleviated in this population. Since the patients with severe and very severe level depression were limited, we did not further divide our patients into different groups according to severity of depression. Therefore, further larger scale, prospective studies are needed to explore extensive information in patients divided according to severity of depression. Third, we used the self-reported history of hypertension and diabetes in the paper, which may underestimate the actual incidence of hypertension and diabetes in the enrolled patients. Fourth, according to Bonferroni correction, the significant of TC as a risk factor for depression should be further considered since the *p* value should be divided by 13 (13 independent factors) in logistic regression analysis.

## Conclusions

In conclusion, NEUT% was significantly and positively correlated with HAM-D_24_ score in hospitalized heart failure patients. Moreover, after the adjustment of confounding factors, the NEUT% is independently associated with depression, it can be used as an new marker for depression in hospitalized heart failure patients. In the future, a simple and cheap NEUT% test may provide insight into depression status and should be included in psychiatric evaluation in hospitalized heart failure patients.

## Abbreviations

BMI: Body mass index
BNP: Brain natriuretic peptide
BUN: Urea nitrogen
CI: Confidence interval
DBIL: Direct bilirubin
FG: Fasting glucose
HAM-D_24_: Hamilton rating scale for depression (24 items)
HDL-C: High-density lipoprotein-cholesterol
LDL-C: Low-density lipoprotein-cholesterol
LVEF: Left ventricular ejection fraction
MPV: Mean platelet volume
NEUT%: Neutrophilic granulocyte percentage
NYHA: New York heart association
PDW: Platelet distribution width
RBC: Red blood cells
TBIL: Total bilirubin
TC: Total cholesterol
TP: Total protein
WBC: White blood cells
